# Optimising medication management in children and young people with ADHD using a computerised test (QbTest): a feasibility randomised controlled trial

**DOI:** 10.1186/s40814-021-00788-1

**Published:** 2021-03-16

**Authors:** Laura Williams, Charlotte L. Hall, Sue Brown, Boliang Guo, Marilyn James, Matilde Franceschini, Julie Clarke, Kim Selby, Hena Vijayan, Neeta Kulkarni, Nikki Brown, Kapil Sayal, Chris Hollis, Madeleine J. Groom

**Affiliations:** 1 School of Medicine, Division of Psychiatry and Applied Psychology, Institute of Mental Health, University of Nottingham Innovation Park, Triumph Road, Nottingham, NG7 2TU UK; 2 Nottingham Clinical Trials Unit, University of Nottingham, Nottingham, UK; 3 Division of Rehabilitation and Ageing, University of Nottingham, Nottingham, UK; 4 United Lincolnshire Hospitals NHS Trust, Grantham and District Hospital, Grantham, Lincolnshire UK; 5 Department of Community Paediatrics, Medway NHS Foundation Trust, Kent, UK; 6 North East London NHS Foundation Trust, Havering CAMHS, Essex, UK; 7 Leicestershire Partnership NHS Trust, Leicester, UK; 8 Centre for ADHD and Neurodevelopmental Disorders (CANDAL), University of Nottingham, Nottingham, UK; 9 NIHR MindTech Medtech Co-operative, Institute of Mental Health, University of Nottingham Innovation Park, Triumph Road, Nottingham, UK; 10 NIHR Nottingham Biomedical Research Centre, Institute of Mental Health, University of Nottingham Innovation Park, Triumph Road, Nottingham, UK

**Keywords:** Attention deficit hyperactivity disorder (ADHD), QbTest, Medication management, Acceptability, Feasibility

## Abstract

**Background:**

Medication for attention deficit hyperactivity disorder (ADHD) should be closely monitored to ensure optimisation. There is growing interest in using computerised assessments of ADHD symptoms to support medication monitoring. The aim of this study was to assess the feasibility and acceptability of a randomised controlled trial (RCT) to evaluate the efficacy of one such computerised assessment, the Quantified Behavior (Qb) Test, as part of medication management for ADHD.

**Methods:**

This feasibility multi-site RCT conducted in child and adolescent mental health and community paediatric settings recruited participants aged 6–15 years diagnosed with ADHD starting stimulant medication. Participants were randomised into one of two arms: *experimental* (QbTest protocol) where participants completed a QbTest at baseline and two follow-up QbTests on medication (2–4 weeks and 8–10 weeks later) and *control* where participants received treatment as usual, including at least two follow-up consultations. Measures of parent, teacher, and clinician-rated symptoms and global functioning were completed at each time point. Clinicians recorded treatment decision-making and health economic measures were obtained. Data were analysed using multi-level modelling and participants (children and parents) and clinicians were interviewed about their experiences, resulting data were thematically analysed.

**Results:**

Forty-four children and young people were randomised. Completion of study outcome measures by care-givers and teachers ranged from 52 to 78% at baseline to 47–65% at follow-up. Participants reported the questionnaires to be useful to complete. SNAP-IV inattention scores showed greater reduction in the intervention than the control group (− 5.85, 95% CI − 10.33, − 1.36,). Engagement with the intervention ranged from 100% at baseline, to 78% follow-up 1 and 57% follow-up 2. However, only 37% of QbTests were conducted in the correct time period. Interview data highlighted that the objectivity of the QbTest was appreciated by clinicians and parents. Clinicians commented that the additional time and resources required meant that it is not feasible to use QbTest for all cases.

**Conclusion:**

The trial design and protocol appear to be feasible and acceptable but could be improved by modifying QbTest time periods and the method of data collection. With these changes, the protocol may be appropriate for a full trial. Adding QbTest may improve symptom outcome as measured by SNAP-IV.

**Trial registration:**

ClinicalTrials.gov, NCT03368573, prospectively registered, 11th December 2017, and ISRCTN, ISRCTN69461593, retrospectively registered, 10th April 2018

**Supplementary Information:**

The online version contains supplementary material available at 10.1186/s40814-021-00788-1.

## Key messages regarding feasibility

1. What uncertainties existed regarding feasibility?

The uncertainties that existed regarding feasibility surrounded the following:

- Whether clinical settings could schedule the additional repeated QbTests and clinical appointments as per the intervention protocol.

2. What are the key feasibility findings?

The key feasibility findings are the following:

- It was difficult for clinics to integrate the additional QbTests and clinical appointments into their current clinical pathway.
- Care-givers were accepting of the protocol and were supportive of attending additional appointments.
- Clinicians viewed the repeated QbTests as positive and beneficial, but wanted to retain autonomy to decide which patients they should offer follow-up QbTests to and how frequently these follow-ups should be conducted, in a future protocol rather than a ‘one size fits all’ approach.
- Time taken between diagnosis and first QbTest to uptake of medication took longer than anticipated which slowed recruitment.

3. What are the implications of the feasibility findings for the design of the main study?

The implications of the feasibility findings surround the need for a more flexible intervention protocol that can work with the varying demand and timescales for scheduling repeated QbTests at clinics, as well as enable clinicians to retain autonomy for which patients would be best supported by additional QbTests to monitor medication outcomes rather than a blanket approach.

## Background

Attention deficit/hyperactivity disorder (ADHD) is a neurodevelopmental disorder, with core symptoms of hyperactivity, inattention, and impulsivity. ADHD affects around 3–5% of children and young people under 18 years old in the UK [1], impacts health-related quality of life [2], impairs academic and social function, and increases risk of substance misuse, unemployment, and mental health problems [3–5]. Furthermore, parenting a child with ADHD can place additional psychological and financial burden on families [6, 7]. Thus, it is essential that children have timely access to evidence-based treatments to reduce symptoms and impairment, support family functioning, and reduce burden on social and healthcare systems [8].

NICE ADHD guidelines [1] recommend children aged over 5 years should be started on methylphenidate as a first-line choice, with treatment tailored to the needs of the individual through frequent monitoring to balance optimal symptom reduction with minimal side effects during titration. Similar recommendations are made by the American Academic of Pediatrics [9], Canadian ADHD Practice Guidelines [10], and the European ADHD Guidelines Group (https://adhd-institute.com/) [11]. A systematic review and network analysis of 133 RCTs found that medications for ADHD, including methylphenidate and amphetamines, are superior to placebo, demonstrating their efficacy in treating the symptoms of ADHD in children and young people [12]. This review also reported that tolerability (measured as study drop-outs due to adverse events) was worse than placebo only for amphetamines and guanfacine in children, suggesting that most ADHD medications are well-tolerated. There is also compelling evidence, however, that the efficacy of ADHD medications and the number of side effects reported are dependent on the child receiving an optimal dose [13] and that carefully monitored dose titration is associated with better clinical outcomes [14]. Audit data from NHS Trusts within the UK indicate that regular monitoring during titration often does not occur [15] and many families are dissatisfied with the service [15, 16]. As a result of time delay to receiving optimal treatment, children may not be fully benefiting from medication, which may impact on their continuing social, societal and academic performance, as well as increase their risk of medication non-adherence. Indeed, 50% of patients have stopped ADHD medication after 18 months and 80% after 3 years [17].

Current practice to evaluate medication effectiveness relies on the clinician integrating the opinions of parents, teachers, the young person, and their own observations, which are often supported by rating scales. Delays in receiving these various sources of information, as well as the subjective and often contradictory nature of the reports, can lead to delays in treatment decisions. To help overcome these difficulties, some UK healthcare clinics have recently incorporated a commercial, objective computerised test of attention, impulsivity, and activity called ‘QbTest’ (Qbtech Ltd, www.qbtech.com), to support clinical decision-making. The test is approved by the FDA (ref: K133382) to be integrated into standard care to support clinical decision-making for diagnosis or medication for ADHD but should not be used as a ‘stand-alone’ test. Briefly, the test combines a computerised test of attention and impulsivity, with an infrared motion capture of head movement to measure activity during the task. The QbTest requires the patient to respond to an infrequently presented stimulus (by pressing a button) but ignore all other stimuli. QbTest provides information on each of the three symptom domains of ADHD (hyperactivity, inattention, and impulsivity) and provides a summary report based on deviation from a normative age and gender data set [18]. Further details on the QbTest have been published [19, 20].

Research has identified the potential utility of the QbTest as a clinical tool to aid ADHD assessment and management [21]. The test has been shown to aid in the differentiation of ADHD from other conditions [22–24], increase clinician confidence in diagnostic decision-making [20], reduce the number of appointments to make a diagnosis [20], and result in cost-savings to health services [25]. Although comparatively less researched than its use for diagnosis, qualitative findings from a recent randomised controlled trial (RCT) indicate the potential for QbTest to aid medication management [26, 27]. Clinician interviews have identified that the QbTest is useful in supporting their confidence in starting medication, choosing suitable titration schedules, communicating to families/schools about medication efficacy, and promoting medication adherence [26]. As well as being valued by clinicians, QbTest has been shown to be efficacious as a tool to assess symptom change as a result of medication. For example, evidence shows the QbTest is sensitive to detecting changes in ADHD symptoms arising from stimulant medication in boys [28], and one study in adults shows that QbTest was more sensitive to medication effects than a standardised rating scale [29]. Another study has shown it may be useful in identifying partial or non-responders after a single dose [30].

Despite growing recognition of the value of QbTest in ADHD assessment and monitoring, there is a lack of evidence as to whether the QbTest should be incorporated into routine practice, whether this would be feasible to do so, and whether this approach would result in improved outcomes. The aim of the QbTest Utility for Optimising Treatment in ADHD’ (QUOTA) study was to develop a medication management protocol for the incorporation of QbTest [19] and test the feasibility and acceptability of the protocol in a feasibility RCT. The findings of the trial will be used to determine whether a future definitive RCT, to test the impact of a QbTest medication monitoring strategy on clinical outcomes, is feasible.

## Methods

### Trial design

**Fig. 1:**
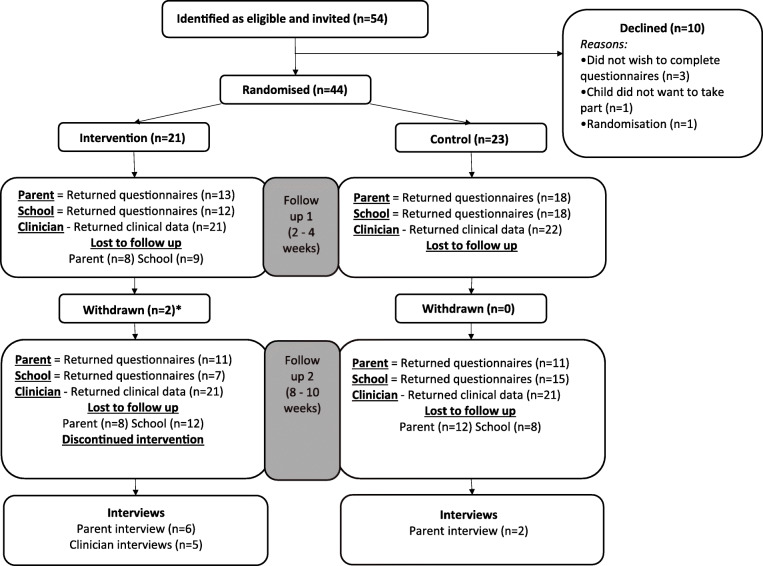
CONSORT flow of participants. *N* = questionnaire with largest return at each time point. Asterisk indicates 1 participant withdrew after completing baseline measures and intervention. 1 withdrew after completing all baseline measures except QbTest

The study was a parallel group, single-blind, feasibility RCT with embedded qualitative evaluation. The study was conducted across five child and adolescent mental health services (CAMHS) or community paediatric clinics in the Midlands and South of England, between December 2017 and October 2018. The protocol, including choice of outcome measures and timing of the interventions were co-designed by patient and public involvement (PPI) members, clinicians, and academics. Full details of the study protocol and the development of its design have been published [19, 31]. The trial was prospectively registered with ISRCTN, trial registration number ISRCTN69461593 and Clinical Trials registration number NCT03368573, and conducted according to CONSORT (Consolidated Standards of Reporting Trials [32]) guidelines for feasibility studies (flow diagram (Fig. 1) and checklist (Additional file 1)).

Since the publication of the initial protocol [19], there has been one change to the trial to extend the recruitment period from July 2018 to October 2018 and increase the number of participating research sites from three to five. These two changes were made in response to lower numbers of eligible participants in the ADHD pathway at the initial sites.

### Participants

Eligible patients were aged 6–17 years, with a clinical diagnosis of ADHD, and about to commence stimulant medication (i.e. methylphenidate or lisdexamfetamine). Exclusion criteria were non-fluent English, unable to provide written consent, or a suspected severe learning disability. No changes to the eligibility criteria were made during the trial. Potential participants were identified, recruited, and consented by either clinicians or clinical research staff at the sites. In line with recommendations for feasibility sample sizes, we aimed to recruit 60 participants (30 per study arm) to test the feasibility and establish preliminary efficacy [33]. All participants were invited to take part in qualitative interviews (see Fig. 1 for participant flow).

The study received ethical approval from the West of Scotland Research Ethics Committee on 7th November 2017, REC ref: 17/WS/0209, and permission was sought from and granted by each National Health Service (NHS) Trust involved in the trial. Written informed consent was obtained—for children aged under 16, written consent was obtained from the parent/legal guardian and verbal or written assent was obtained from the child/young person.

### Interventions

#### Experimental arm (QbTest medication monitoring)

The QbTest medication monitoring intervention added QbTest to treatment as usual (TAU). QbTests were completed at baseline, and then 2 to 4 weeks later. At each time point, the clinician reviewed QbTest results along with other clinical tools to monitor medication (follow-up 1) and repeated this procedure again at eight to 12 weeks (follow-up 2).

#### Control arm

TAU differs across sites; to ensure some standardisation of practice in this trial, participants received their site’s standard TAU. If this did not already include two contacts within a 12-week period following medication initiation, the sites were asked to add two contacts with the consultant psychiatrist or paediatrician by the end of the 12-week study period. These contacts were either in person or by telephone dependent upon the consultant’s preference. This was designed to ensure both study arms received a similar level of clinical contact during the study period so that any differences between arms in outcomes were more likely due to the addition of QbTest rather than overall amount of clinical contact.

In both arms, the participant’s usual care team were responsible for patient care and administering the QbTests. Full details of the intervention and TAU have been previously published in the protocol [14].

### Randomisation and blinding

Participants were randomised on a 1:1 ratio into either the QbTest medication arm (experimental arm) or control arm, using block randomisation stratified by site, via sealed opaque envelopes. The clinicians or clinical research staff were not blinded to allocation once randomisation had occurred, but outcome assessors were blind to arm allocation. Choice of medication did not have to be selected prior to randomisation.

### Measures

#### Feasibility and acceptability

Data were collected on a range of feasibility and acceptability measures. These included the number of participants randomised, number of people declining to take part (along with reasons where given), withdrawals of participants post-randomisation, number of outcome measures completed by parents, children, and schools at each time point, number of clinician pro formas (see below) completed, number of QbTests conducted, and participant and clinician opinions through interviews.

#### Between-group outcome measures

Parents and carers were given the choice of completing outcome measures online, via telephone, or post. Clinicians and teachers had the option of online or postal forms. All participants and schools were included at each follow-up regardless of previous missing data, unless they withdrew from the study. When self-report questionnaires from schools and families were not returned after two weeks, they were re-sent and followed up via phone or email.

As this was a feasibility RCT, there was no specified primary outcome. A full description of each measure can be found in the protocol [19]. Details on the validated data collection tools used to address the study objectives can be found in the protocol [19], briefly the measures were as follows:

*Swanson, Noland and Pelham version IV (SNAP-IV)* [34]. A brief behavioural rating scale that measures core symptoms ADHD and oppositional defiant disorder (ODD), rated by parents and teachers

*Strengths and Difficulties Questionnaire (SDQ)* [35, 36]. A brief measure of behavioural and emotional difficulties rated by parents and teacher

*Child Health Utility 9 Dimensions (CHU9D)* [37]. Completed by either the young person or parent to provide a paediatric generic preference-based measure of health-related quality of life

*QbTest* [18]. A 20-min computer test of attention and impulsivity with a measure of activity, completed by the young person

*Clinical Global Impressions Scale (CGI)* [38]. A brief measure of overall severity and improvement in symptoms as rated by the clinician

*Side effects scale* [39]. A brief measure of common anticipated side-effects in children with ADHD

Additional data collection tools were developed specifically for the study, and a medication adherence questionnaire, a clinician pro forma, and study-specific health economics questionnaires for participants and schools. The medication adherence questionnaire is a short form that allows the parent/carer to record the medication and dose being taken by the child and how regularly it is taken. The clinician pro forma records data on diagnoses and clinical decision-making along with the resources used to reach decisions. Two health economics questionnaires were designed specifically for the study and examined the inventory provided by Client Service Receipt Inventory CSRI [40] but focusing on meaningful variables for ADHD. One questionnaire titled ‘Resource Use – Services for Education’ (RUSE) was designed to collect data on education resources the child uses and was completed by teachers. The other titled ‘Resource Use – Services for Health’ (RUSH) was designed to be completed by parents/carers to gather data relating to health services used by families for the child, along with any impacts on family life, such as time out of employment to care for the child. In this study, we sought to assess the feasibility and acceptability of these new measures only; health economics values are not reported. The time points when each measure was collected are shown in Table 2.

#### Qualitative interviews

All intervention and control participants, alongside a parent or guardian, were invited to partake in telephone interviews with a researcher (LW) to discuss their experience of QbTest; clinicians were also invited to take part in a telephone interview. Interview schedules for participants and clinicians explored—from their different perspectives—the experience of the study and the acceptability of QbTest for its intended purpose. Schedules included questions aiming to uncover barriers and facilitators relating to the protocol’s implementation, engagement with the technology, and its impact on care. Within the clinician interviews, normalisation process theory (NPT) [41] informed part of the schedule in order to explore the potential future implementation of QbTest. The domains of NPT are coherence (understanding around the purpose of QbTest), cognitive participation (engagement with the QbTest as part of care), collective action (activities that people do to implement it), and reflexive monitoring (any reviewing or appraisal of the impact of QbTest).

### Analysis

#### Quantitative

Descriptive statistics (mean and standard deviation) were summarised by group and across follow-up time (if applicable) for each outcome measure. Preliminary treatment effects were examined using multi-level modelling and are presented together with their 95% confidence intervals. Recruitment rate, retention rate, and completion rates of each measure were calculated from the data. All statistical analysis was conducted using STATA15.

#### Qualitative

The qualitative interviews were audio recorded, transcribed verbatim, and analysed thematically following the guidelines of Braun and Clarke [42]. Qualitative software, NVivo 12, was used for coding and management of data. Analysis was conducted using pre-defined areas of inquiry relating to the primary research questions; researchers focused on content in the following areas: acceptability of the protocol (and whether any adjustments should be made), desirability of QbTest as part of medication management, and for clinician interviews, the domains of NPT were explored to provide early insight into its potential future implementation. Throughout the process, researchers were also attentive to data that was unexpected, which was subsequently explored to decide whether it was of interest to the aims of the study. Thus, the thematic analysis was mainly driven by a particular set of aims, but allowed for inductively generated (unexpected) themes.

## Results

### Feasibility and acceptability

#### Recruitment and randomisation

The first participant was enrolled on 18th December 2017 and recruitment ended on 30th October 2018. The last participant exited the trial on 30th December 2018 when the trial ended.

In total, 54 participants were deemed eligible by clinicians across the five sites and invited to participate in the study. Demographics and clinical characteristics of participants at baseline by randomised group are presented in Table 1. Randomisation was evenly split across the study arms. Ten participants eligible for inclusion declined to take part (see Fig. 1). Two participants withdrew from the intervention arm after baseline assessment (see Fig. 1).

**Table 1:** Participant demographics and clinical characteristics

		Intervention (*n* = 21)	Control (*n* = 23)
Mean (SD) age in years when entered the study		9.29 (2.81)	9.22 (2.19)
		*n* (%)	*n* (%)
Gender	**Male**	20 (95.24)	19 (82.61)
	**Female**	1 (4.76)	4 (17.93)
Ethnicity	**Bangladeshi**	1 (4.76)	0
	**Dual heritage**	1 (4.76)	0
	**Not given**	1 (4.76)	0
	**Other**	1 (4.76)	1 (4.35)
	**Pakistani**	1 (4.76)	1 (4.35)
	**White**	16 (76.19)	21 (91.30)
ADHD diagnosis	**ADHD combined subtype**	18 (85.71)	14 (60.87)
	**ADHD—hyperactive-impulsive**	2 (9.52)	5 (21.75)
	**ADHD—inattentive**	1 (4.76)	2 (8.70)
	**Dual diagnosis ASD**	0	1 (4.35)
Other clinical diagnoses	**ASD/social communication/speech difficulties**	3 (14.28)	5 (21.75)
	**Attachment disorder**	0	1 (4.35)
	**Conduct disorder**	0	2 (8.70)
	**Tic and neurological disorders**	2 (9.52)	0
	**Mood disorders**	1 (4.76)	0

Data presented as *n* (%) unless otherwise specified. *ADHD* Attention deficit hyperactivity disorder, *ASD* Autism spectrum disorder

Of five sites, one returned detailed information about screening, eligibility, and recruitment. At this site, of 41 children identified as eligible, 11 were not approached due to lack of clinic resources, eight families decided not to start taking medication or the clinician decided not to start medication then, eight did not want to take part in the study, and four were initially interested but started medication before a baseline test could be carried out. This left a sample of 10 participants recruited at that clinic over a 4-month period (25% recruitment rate).

#### Engagement with the intervention

Completion rates for questionnaire outcome measures and QbTest decreased across the time points in each arm, for each group (see Table 2).

**Table 2:** Measure completion rates at baseline, follow-up 1, and follow-up 2 for the intervention and control group

Outcome measure	Intervention *n* (%)			Control *n* (%)		
	Parents	School	Clinician	Parents	School	Clinician
SNAP-IV						
*Baseline*	10 (52)	12 (63)	-	18 (78)	18 (78)	-
*Follow-up 1*	11 (57)	15 (78)	-	12 (52)	15 (65)	-
*Follow-up 2*	10 (52)	9 (47)	-	11 (47)	15 (65)	-
SDQ						
*Baseline*	13 (68)	12 (63)	-	16 (69)	18 (78)	-
*Follow-up 2*	9 (47)	9 (47)	-	11 (47)	15 (65)	-
CHU9D						
*Baseline*	12 (63)	-	-	16 (67)	-	-
*Follow-up 1*	10 (52)	-	-	11 (47)	-	-
*Follow-up 2*	9 (47)	-	-	11 (47)	-	-
Medication Adherence Questionnaire						
*Follow-up 1*	11 (57)	-	-	11 (47)	-	-
*Follow-up 2*	10 (52)	-	-	11 (47)	-	-
Side effects Questionnaire						
*Follow-up 1*	11 (57)	-	-	11 (47)	-	-
*Follow-up 2*	10 (52)	-	-	11 (47)	-	-
RUSH						
*Follow-up 2*	10 (52)	-	-	11 (47)	-	-
RUSE						
*Follow-up 2*	-	9 (47)	-	-	13 (56)	-
QbTest						
*Baseline*	19 (100)	-	-	19 (82)	-	-
*Follow-up 1*	15 (78)	-	-	-	-	-
*Follow-up 2*	11 (57)	-	-	-	-	-
Clinician Proforma						
*Baseline*	-	-	21 (100)		-	22 (95)
*Follow-up 1*	-	-	21 (100)	-	-	21 (91)
*Follow-up 2*	-	-	18 (94)	-	-	20 (87)
*CGI*						
*Baseline*	-	-	20 (95)	-	-	21 (91)
*Follow-up 1*	-	-	-	-	-	-
*Follow-up 2*	-	-	17 (81)	-	-	20 (87)

*CGI* Clinical global impression scale, completed by clinician at baseline and follow-up 2; *CHU9D* Child Health Utility 9D, completed by parents or child at baseline, follow-up 1, and 2; *SNAP-IV* Swanson, Noland and Pelham IV Questionnaire, completed by parents and teachers at baseline, follow-up 1, and 2; *SDQ* Strengths and Difficulties Questionnaire, completed by parents and teachers at baseline and follow-up 2; *Medication adherence* completed by parents at follow-up 1 and 2; *Side effects* completed by parents at follow-up 1 and 2; *RUSH* Resource Use for Health, completed by parents at follow-up 2; *RUSE* Resource Use for Education, completed by teachers at follow-up 2; *QbTest* Conducted at baseline for control group and at baseline, follow-up 1, and 2 for intervention group. *Proforma* clinician proforma completed by clinicians at baseline, follow-up 1 and 2

Protocol deviations were noted in each arm. In the intervention group, 37% (7/19) of follow-up 1 assessments were completed within the 2–4-week timeframe specified in the study protocol, and 53% (10/19) completed follow-up 2 within the 12-week timeframe. Information gained from the clinician completed pro forma in the intervention arm found that across both follow-ups, 73% (24/33 clinician responses) found QbTest was useful in determining their treatment, 18% (6) were neutral, and 9% (3) stated it was not helpful. A further inspection revealed that more clinicians tended to find it helpful at follow-up 1 (76.5%, 13/17) than follow-up 2 (68.8%, 11/16).

In the control arm, the study protocol required two contacts any time within the 12 week study period: 70% (16/23) of control participants had one contact with the clinical team within 12 weeks and 30% (7/23) had two contacts within 12 weeks. No serious adverse events were reported and the side effects scores decreased in both arms from T1 to T2.

### Between-group outcome measures

**Fig. 2:**
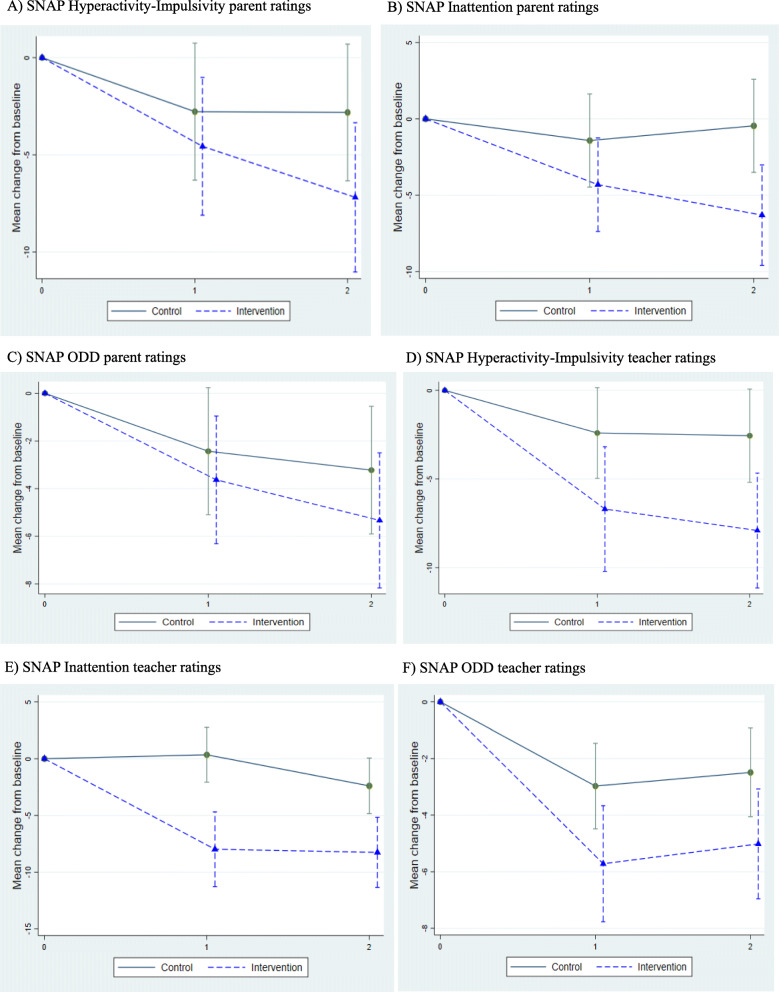
Modelled mean change (95% CI) on SNAP scores from baseline. Data shown are the mean and 95% confidence interval change from baseline at each time point on the SNAP-IV in the control and intervention groups. **a**–**d** Parent and teacher ratings on the hyperactivity-impulsivity and inattention subscales

All results from the between-group outcome measures are presented in Table 3 and changes in outcome scores, along with 95% confidence intervals, are provided in Table 4 for all measures except the CHU9-D. Data analysis was not performed on the CHU9D as initial checks on the data revealed it was not meaningful to combine data from parent and child respondents, resulting in sample sizes that were too small for analysis. For all scales, higher scores represent greater presence of symptoms/impact. For the CGI, higher scores represent greater improvement. Figure 2 shows the difference between study arms over time on the SNAP-IV parent and teacher scales and indicates greater improvement in change scores in the intervention arm than the control.

**Table 3:** Mean scores (SD) on outcome measures for the intervention and control groups

Completer	Outcome measure	Baseline		Follow-up 1		Follow-up 2	
		Intervention	Control	Intervention	Control	Intervention	Control
**Parent**	**SNAP-IV**	*n* = 10	*n* = 16	*n* = 11	*n* = 12	*n* = 10	*n* = 11
	ODD	14.69 (4.96)	14.69 (6.69)	10.82 (6.75)	12.17 (7.30)	10.20 (6.01)	9.55 (5.66)
	Hyp/Imp	19.46 (6.08)	20.75 (6.45)	14.64 (7.74)	17.92 (6.60)	12.80 (6.86)	15.33 (6.93)
	Inattention	16.4 (5.14)	19.88 (4.30)	15.82 (5.74)	18.08 (5.58)	13.30 (5.36)	17.45 (5.54)
	**SDQ**	*n* = 13	*n* = 16			*n* = 10	*n* = 11
	Emotional problems	4.08 (2.60)	4.50 (2.66)	-	-	4.00 (3.23)	4.52 (2.03)
	Conduct problems	4.62 (2.57)	4.25 (2.14)	-	-	3.50 (2.22)	3.36 (1.57)
	Hyperactivity	8.46 (2.37)	9.06 (1.06)	-	-	7.00 (2.67)	7.43 (1.80)
	Peer problems	4.15 (2.30)	4.75 (2.11)	-	-	3.60 (1.78)	4.62 (2.86)
	Prosocial	6.46 (2.37)	6.06 (1.98)	-	-	5.90 (2.42)	6.75 (1.49)
	Total difficulties	21.31 (6.98)	22.56 (5.29)	-	-	18.10 (7.50)	19.92 (5.75)
	Externalising	13.08 (4.54)	13.31 (2.70)	-	-	10.50 (4.55)	10.79 (3.20)
	Internalising	8.23 (4.44)	9.25 (3.87)	-	-	7.60 (4.14)	9.13 (4.51)
	Impact	4.62 (2.96)	5.27 (2.71)	-	-	3.78 (2.22)	3.94 (3.37)
	**Side effects scale**			*n* = 11	*n* = 10	*n* = 10	*n* = 11
		-	-	21.29 (11.31)	18.08 (9.84)	20.80 (14.34)	16.55 (10.87)
	**CHU9D**	*n* = 8	*n* = 8	*n* = 6	*n* = 7	*n* = 6	*n* = 7
		24.75 (6.25)	22.88 (9.58)	20.83 (4.54)	19.92 (9.52)	21.83 (8.75)	15.57 (3.99)
**Child**	**CHU9D**	*n* = 5	*n* = 7	*n* = 9	*n* = 6	*n* = 3	*n* = 4
		21.20 (7.69)	19.14 (5.67)	19.79 (5.33)	18.17 (5.04)	26.29 (13.69)	29.00 (2.16)
**Teacher**	**SNAP**	*n* = 13	*n* = 18	*n* = 8	*n* = 15	*n* = 9	*n* = 15
	ODD	7.92 (6.44)	9.44 (6.87)	3.25 (2.12)	7.47 (4.47)	4.33 (4.56)	8.18 (5.72)
	Hyp/Imp	10.62 (7.57)	14.72 (8.34)	4.75 (2.25)	12.67 (6.56)	4.44 (5.73)	11.93 (6.30)
	Inattention	12.08 (7.37)	17.28 (7.54)	7.00 (5.13)	16.87 (6.21)	6.22 (5.31)	14.13 (5.57)
	**SDQ**	*n* = 11	*n* = 18			*n* = 9	*n* = 15
	Emotional problems	3.00 (3.29)	3.39 (2.93)	-	-	1.56 (1.33)	3.20 (2.46)
	Conduct problems	3.00 (2.93)	2.61 (2.17)	-	-	1.00 (1.12)	2.93 (2.43)
	Hyperactivity-inattention	6.82 (2.68)	8.33 (1.91)	-	-	3.44 (2.19)	7.60 (2.32)
	Peer problems	2.09 (1.81)	3.56 (2.04)	-	-	2.00 (1.50)	3.67 (2.13)
	Prosocial	5.09 (2.66)	6.06 (2.48)	-	-	6.67 (2.35)	5.80 (2.21)
	Total difficulties	14.91 (7.02)	17.89 (5.97)	-	-	8.00 (3.35)	17.40 (6.25)
	Externalising	9.82 (5.40)	10.94 (3.08)	-	-	4.44 (3.09)	10.27 (4.25)
	Internalising	5.09 (3.62)	6.94 (4.41)	-	-	3.56 (1.51)	6.87 (3.70)
	Impact	2.09 (1.64)	3.22 (2.05)	-	-	0.75 (1.16)	1.67 (1.61)
**Clinician**	**CGI**	*n* = 20	*n* = 21	-	-	*n* = 17	*n* = 20
	Severity	3.65 (0.98)	3.67 (1.32)	-	-	2.06 (1.14)	2.10 (1.23)
	Improvement	-	-	-	-	1.82 (0.88)	2.37 (1.01)

Data are mean (1 SD). *n* sample size for analysis; *CGI* Clinical Global Impression, baseline and follow-up 2; *CHU9D* Child Health Utility 9D, baseline, follow-up 1, and 2; *Hyp/Imp* Hyperactivity/impulsivity; *ODD* Oppositional defiant disorder; *SDQ* Strengths and difficulties questionnaire, completed by parents and teachers at baseline and follow-up 2; *SNAP-IV* Swanson, Noland and Pelham IV Questionnaire, completed by parents and teachers at baseline, follow-up 1, and 2

**Table 4:** Mean change in scores and difference between the intervention and control group

Rater	Outcome measure	Follow-up 1			Follow-up 2		
		Intervention	Control	Mean difference	Intervention	Control	Mean difference
Parent	**SNAP-IV**						
	Inattention	− 4.31 (− 7.37, − 1.25)	− 1.42 (− 4.47, 1.63)	− 2.89 (− 7.21, 1.43)	− 6.30 (− 9.59, − 3.01)	− 0.46 (− 3.51, 2.59)	− 5.85 (− 10.33, − 1.36)
	Hyp/Imp	− 4.56 (− 8.11, − 1.02)	− 2.78 (− 6.30, 0.75)	− 2.78 (− 6.30, 0.75)	− 7.19 (− 11.03, − 3.35)	− 2.82 (− 6.34, 0.70)	− 4.37 (− 9.58, 0.84)
	ODD	− 3.63 (− 6.31, − 0.95)	− 2.43 (− 5.10, 0.24)	− 1.20 (− 4.98, 2.58)	− 5.33 (− 8.17, − 2.50)	− 3.22 (− 5.90, − 0.55)	− 2.11 (− 6.01, 1.80)
	**SDQ**						
	Hyp/Inatten	-	-	-	− 2.22 (− 3.90, − 0.53)	1.16 (− 2.67, 0.35)	− 1.06 (− 3.32, 1.20)
	Tot score	-	-	-	− 5.26 (− 8.23, − 2.28)	− 1.18 (− 3.84, 1.48)	− 4.08 (− 8.07, − 0.09)
	Impact	-	-	-	− 0.74 (− 2.89, 1.41)	− 0.71 (− 2.60, 1.19)	− 0.03 (− 2.90, 2.83)
	**Side effects**	22.12 (16.02, 28.22)	16.44 (10.10,22.77)	5.69 (− 3.10, 14.47)	19.34 (13.09, 25.59)	16.70 (10.51, 22.89)	2.64 (− 6.15, 11.43)
Teacher	**SNAP-IV**						
	Inattention	− 5.72 (− 7.77, − 3.67)	− 2.97 (− 4.48, − 1.46)	− 2.74 (− 5.30, − 0.18)	− 5.02 (− 6.96, − 3.08)	− 2.49 (− 4.05, − 0.92)	− 2.53 (− 5.04, − 0.02)
	Hyp/Imp	− 6.69 (− 10.21, − 3.18)	− 2.40 (− 4.96, 0.15)	− 4.29 (− 8.72, 0.14)	− 7.91 (− 11.15, − 4.67)	− 2.56 (− 5.19, 0.07)	− 5.35 (− 9.55, − 1.14)
	ODD	− 7.98 (− 11.28, − 4.68)	0.35 (− 2.06, 2.76)	− 8.33 (− 12.56, − 4.10)	− 8.26 (− 11.35, − 5.17)	− 2.39 (− 4.84, 0.06)	− 5.87 (− 9.89, − 1.85)
	**SDQ**						
	Hyp/Inatten	-	-	-	− 3.31 (− 4.72, − 1.90)	− 0.65 (− 1.71, 0.41)	− 2.66 (− 4.47, − 0.84)
	Total score	-	-	-	− 6.75 (− 9.96, − 3.53)	− 0.82 (− 3.24, 1.61)	− 5.93 (− 10.05, − 1.81)
	Impact	-	-	-	− 2.16 (− 3.07, − 1.25)	− 1.78 (− 2.53, − 1.04)	− 0.38 (− 1.62, 0.86)

Data shown are the mean change from baseline (with 95% confidence interval) at each time point in each trial arm and the mean difference between arms (with 95% CI) and associated *p* value. *SNAP-IV* Swanson, Noland and Pelham IV Questionnaire, completed by parents and teachers at baseline, follow-up 1, and 2; *SDQ* Strengths and Difficulties Questionnaire, completed by parents and teachers at baseline and follow-up 2; *Hyp/Imp* Hyperactivity/impulsivity; *ODD* Oppositional defiant disorder

At follow-up 1, 55.6% (10/18) of cases in the experimental arm underwent changes to their treatment plan in comparison to only 33.3% (7/21) of cases in the control arm. At follow-up 2, the figures were more comparable with slightly fewer changes planned in the intervention arm (41.2%, 7/17) in comparison to the control (47.4%, 9/19). In all instances, changes to the treatment plan involved changes to the type or dose of ADHD medication.

A comparison of medication adherence scores between the two arms revealed similar results. For those that responded, at follow-up 1, 100% (8/8) in the experimental arm and 88.8% (8/9) in the control arm reported taking their medication most/every day. At follow-up 2, these figures were 87.5% (7/8) in the experimental arm and 100% (9/9) in the control arm.

### Qualitative data on acceptability of the protocol and of QbTest as part of care

Eight participants agreed to be interviewed (six from the intervention group and two from the control group). All interviews were conducted with the children and young people’s primary care- givers; whilst it was initially intended that children would also be interviewed alongside their parents, this proved difficult in the initial interviews due to the young age of participants, and so the focus shifted to interviewing parents only. The characteristics of the children whose parents/primary care-givers took part in an interview are presented in Table 5. Five clinicians (from 4 of the 5 clinic sites) agreed to be interviewed, four of which were community paediatricians and one was a psychiatrist; all were female.

**Table 5:** Characteristics of child participants whose parents/carers were interviewed

ID	Age of child (years)	ADHD diagnosis	Any other diagnosis	Site	Study arm
P1	6	ADHD combined subtype	No	1	Intervention
P2	11	ADHD combined subtype	No	3	Intervention
P3	12	ADHD combined subtype	No	1	Intervention
P4	6	ADHD combined subtype	Yes	1	Intervention
P5	9	ADHD combined subtype	Yes	4	Intervention
P6	9	ADHD combined subtype	No	4	Intervention
P7	8	ADHD combined subtype	Yes	1	Control
P8	9	ADHD predominantly inattentive	No	2	Control

*ADHD* Attention deficit hyperactivity disorder

The interviews showed a range of challenges and benefits of using the QbTest as part of medication management. Attention was paid to what aspects of the protocol might require amendment and which aspects were acceptable, in order to inform any subsequent trial design for a full RCT. The themes presented show the acceptability of taking part in the trial and highlights how the test fitted (or did not fit) within everyday care.

#### Theme 1: Acceptability of being part of a study

Separate to having QbTest form part of a person’s care, it is also important to understand the acceptability of trial procedures to families and children. This includes core elements such as randomisation and completion of multiple questionnaires. It was therefore crucial to understand how this was experienced by families.

##### Randomisation and recruitment

None of the participant interviews indicated concerns with randomisation and all understood they may be allocated to either study arm. Those in the intervention arm were pleased to have been randomised into that group and discussed perceived benefits of being randomised into the QbTest arm, notably, the opportunity to have additional QbTests.

Yeah I was fine! It’s like, sort of like, 50/50, so does it make any difference!—P8

It didn’t bother me at all really. Because I mean obviously, we’ve had that additional care there with him having the three QbTests, which, as I say has been helpful for us when we noticed there was a change in his behaviour. So, you know, I was quite happy that we were put into that category.—P1

Those in the control group also felt that their telephone consultations were helpful and indicated that there was some benefit from being in the TAU arm because this required less effort than the three QbTests in the intervention arm.

Yeah, yeah, it worked really well, because it was much easier than having to try and lug him there, and obviously it didn’t disrupt his routine or anything like that as well.—P7

##### Completing questionnaires

Parents had no reservations about completing questionnaires and found them useful in highlighting areas of improvement or change, albeit noting there were multiple questionnaires.

Yeah, as I say, we noticed improvements, the forms are obviously standardised and repetitive, but yeah you can sort of see, you know, you’re making the change obviously as you’re noticing the behavioural change. So yeah, they were absolutely fine.—P1

#### Theme 2: QBTest as part of care

##### Factoring in multiple appointments

Having QbTest as part of care meant that additional appointments were required in order to conduct tests and review results. This could provide both a challenge (in terms of additional burden) and also benefit (in terms of additional care for families, and quicker medication titration). The impact on both clinicians and families will be considered.

Scheduling three appointments within 12 weeks posed some logistical challenges for clinicians and services. The protocol required them to schedule appointments within shorter timeframes than demand on the clinics would ordinarily allow. Other considerations surrounded the use of clinic time in close succession when they might not have considered it necessary to have an appointment to monitor the medication so soon after the last appointment.

For one, our clinic time is very precious so we don’t have the luxury of seeing families so close, erm, together in appointments, and also I am not sure if that is clinically required.—C3

In contrast, the primary care-givers valued the short time frame and described it as beneficial to them and their families.

I think it has been good to have that support. Because, when we have been in the past, we have had an appointment and then not been seen for months and months. P3

The primary care-givers in this sample were satisfied with the study’s appointment schedule, but they had wider concerns regarding the time out of school that clinical appointments and QbTests required, which were not limited to the study, but also related to their overall ADHD care pathway. Whilst it was discussed that appointments before school, after school, and in school holidays would be preferable to missing school, ultimately attending the appointments was considered beneficial.

I would have been fine, because it was for my child’s welfare, and so, you know, I would be quite happy to go to the appointment. But these appointments are all set out for before school or during school, so it is always missing education time, … so I wish they made, you know, appointments in June half term, or after-school appointments, or something like that. It would be helpful.—P5

The clinicians also noted that clinic appointments often occur during working hours which has implications for children and young people missing school and also requires primary care-givers to take time out of work. Running multiple QbTest appointments could increase these problems.

Do children want to come in on a Saturday morning? Probably not! But parents, parents might find it quite useful because they are not necessarily having to take time off work.—C5

##### Avoiding unnecessary workload

Whilst the implementation of QbTest undoubtedly meant conducting additional tests, clinicians voiced a preference to do this only when it was perceived to add value. Clinicians raised concerns about the requirement to conduct QbTests on all of the intervention participants. They described QbTest as one of a suite of tools they use to monitor ADHD symptoms and felt that the additional resources required to carry out QbTests (staffing, clinic time, and test interpretation) are not necessary in routine cases but may be of use in trickier cases. Some also considered repeat QbTests to be burdensome for the children and young people.

… I wouldn’t think it is a great use of resources for every child with ADHD to have it in order to monitor their medication. I think it would be more helpful for the more complicated children who had, you know, other things going on as well, and where the situation wasn’t entirely clear.—C4

The second follow up in the experimental arm was too much, especially for the children. A lot of the children did not want to do the QbTest, or they have one for the assessment, one for the follow up and one for the second, and, you know, some children are compliant and will just do as they are asked to do, but some really didn’t and said ‘I don’t want to do it.—C3

An additional point raised by clinicians was that a greater number of participants could have been eligible to take part in the study if the time window of the baseline QbTest was increased. The study protocol stipulated that a QbTest conducted within 12 weeks prior to inclusion in the study could be used as the baseline QbTest. Any QbTest conducted prior to this previous 12 weeks was considered unreliable, due to potential changes in symptoms, and a new QbTest was required. Clinicians felt that this time window could be increased to widen eligibility and that this would be more in keeping with their clinical practice, where patients with a QbTest older than 12 weeks would not be requested to repeat the test before medication was prescribed.

#### Theme 3: Confidence and communication

QbTest was described by both groups (parents and clinicians) as increasing their confidence in the child’s treatment. Whilst clinicians did not perceive it as necessary in all cases, they found it particularly helpful in more complex cases (as described above).

Primary care-givers considered the repeated QbTests to be useful in increasing confidence in ongoing medication decisions as well as a tool the clinicians used to communicate changes in ADHD symptoms. They did not view the QbTests as burdensome to the children and young people, but they were considered ‘boring’ by some of the children who undertook them.

Yes, because I brought them home and showed my partner as well, and I have been able to explain to him what they mean and he can understand it as well, and we have been able to see the difference on that. It might just be a scribble on a piece of paper, but we can see that there is a difference.—P3

He can have as many of them as they want to give him, it don’t hurt him so, it’s only 15 minutes of his life it’s not going to kill him.—P2

Both groups voiced the potential of QbTest as an aid to communication. Parents described above how a visual representation of their child’s symptoms helped them to better understand the impact of treatment. For clinicians, similar views were expressed, and they also describe it as giving extra weight to their advice during consultations. Therefore, whilst the QbTest is both an aid to communication, it is also a powerful additional ‘voice’ in the discussion. This appears to help parents be more accepting of treatment recommendations.

There are times when families say it [medication] doesn’t work because they are focusing on the behavioural problems which I do not expect the medication to make a huge impact on…so I use the QbTest as a way to talk to them and say ‘look changing the medication or increasing the medication is not going to make a huge difference to the behaviour which we need to address in a different way, so the QbTest is often quite helpful to have those sort of conversations.—C3

## Discussion

The aim of this study was to assess the feasibility and acceptability of adding QbTest to treatment as usual to monitor medication outcomes in ADHD after initiation of stimulant medication [19, 31]. The study protocol was tested in a feasibility RCT and primary care-givers and clinicians involved in the study were interviewed. The findings indicate that the protocol was generally considered acceptable to primary care-givers and clinicians but that some changes are required to improve participant recruitment rates and the feasibility of implementing the protocol within clinical services. These issues were discussed at a stakeholder workshop held immediately after the end of participant recruitment, attended by 16 stakeholders, including representation from each clinic site, the core research team, and PPI members [31]. The aim of the workshop was to identify potential barriers to implementing the protocol and possible ways to refine the protocol to overcome these barriers in a future trial. We now discuss the main findings from the study and will make reference to the comments made by stakeholders at this workshop, throughout.

### Feasibility and acceptability of recruitment and randomisation

Recruitment of participants was more difficult than anticipated and the final sample size of 44 was below the target sample size of 60. During interviews, it became clear that the recruitment difficulties were more strongly related to the feasibility of implementing the study protocol within clinical services than the acceptability of the protocol to potential participants. Only one potential participant declined to take part due to randomisation and three declined to take part because they felt completion of the outcome measures would be too burdensome. None of the parent/carers who were interviewed considered randomisation to be negative and, when further questioned, understood they may be allocated to either study arm and were comfortable with this. Conversely, whilst clinicians commented positively on the QbTest in interviews, they highlighted some issues with the feasibility of the protocol. Specifically, the clinicians felt that the requirement that a baseline QbTest must have been completed within 12 weeks prior to medication initiation limited the numbers of eligible participants. This requirement also did not fit with usual practice where QbTest results older than 12 weeks are often used as a baseline prior to medication initiation. The clinicians felt that this inadvertently excluded families who were returning to the pathway after taking some time to make a decision about whether to commence medication. Although in the current protocol participants could complete a new baseline QbTest at the point of medication initiation, the reality of scheduling this test would have meant a delay in starting medication, which would have been unethical. In the future, expanding the timeframe for existing QbTests would increase the number of families eligible to be approached to take part in the study.

### Outcome measures

The completion and return of study outcome measures by primary care-givers and teachers was similar across measures, although returns by teachers were affected by the timing of school holidays, particularly the long (6-week) summer holidays. Our response rates were lower than we would anticipate based on similar trials [27]; however, during interviews, the primary care-givers acknowledged that although the trial included several questionnaires, they were not unduly burdened by completing them. Based on our previous experience of successful trials, we would implement the following strategies to facilitate completion rates: conduct measures online with researcher telephone support where needed, including out-of-hours telephone appointments, and provide personalised thank you messages when measures are complete. The completion of study measures by clinicians was also high but, during interviews, clinicians commented that the clinician pro forma (developed specifically to enable clinicians to record clinical decision-making within this study) needs to be refined to more reflective of clinician practice and decision-making.

One aim of this study was to identify a primary outcome measure for a future RCT. The SNAP-IV mean scores and change in mean scores, as well as difference in this change between arms, suggests this would be a useful primary outcome to measure the clinical utility of adding QbTest to medication management for ADHD. Furthermore, the return rate for this measure was comparable with the other measures, even though the requirement was for parents and teachers to complete it at all time points. Additionally, parents and clinicians in our previous workshops identified changes on the SNAP-IV as being the most meaningful outcome [43]

Health economics questionnaires, RUSH and RUSE, were designed specifically for the study, and examined the inventory provided by CSRI [40] but focusing on meaningful variables for ADHD. The measures were co-designed by an expert in health economics (MJ), experts in ADHD (MG, KS, CH) and PPI representatives. Primary care-givers and teachers were asked to complete the measures at the second follow-up. The return rates were similar to the other measures, but some items were unanswered, suggesting that respondents either did not know how to answer some questions or felt they were not relevant. Amendments to these measures will be required before they can be used to assess service use in a full RCT. In addition, the CHU9D, a measure of child health utility that is used in combination with life-years to calculate quality-adjusted life-years (QALYs), can be completed by either the child or primary care-giver. Repeated measures are crucial to establish a change in health over time, but there is a requirement that the same respondent completes the measure at each time point. In this study, a mixture of parents and children completed the measure at different time points, rendering the data unsuitable for analysis. To enhance the feasibility of this measure in a future trial, a prior decision should be made as to whether the parent/carer or child should complete the measure, and this should be communicated clearly to families.

Although medication adherence was comparable across both groups, it is noteworthy that our sample was young (mean age 9 years), whereas research shows that evidence of medication effectiveness is important to increase adherence in older teenage children [44], a further trial including older children would be needed to fully assess this. It is also noteworthy that medication adherence was relatively high in general, which may be because the follow-up period was short (maximum 10–12 weeks).

Participants were given a choice between completing their outcome measures via post, online, or over the phone. The majority requested post but, when questioned, stated that they would have been willing to complete questionnaires online. This would be a more cost-effective and pragmatic approach in the future with the option of postal returns reserved only for families with no internet access. In addition, participants in the intervention arm could be given the option of completing outcome measures at their follow-up appointments to increase completion rates.

### Engagement with intervention and protocol adherence

Engagement with the intervention was high amongst participants. Only two participants withdrew from the intervention group at baseline and only one of the remaining participants did not attend their clinic appointments. Interview data reflected positive engagement from the families. Although during interviews the clinicians raised concerns about the burden of multiple QbTests for the children and young people, parents raised no concerns about attending three QbTests in 12 weeks and did not seem to feel that this was burdensome to the child. However, the requirement for the child to take time out of school to attend healthcare appointments was viewed negatively by parents and care-givers. These comments were not specific to the appointments required by the study protocol but reflective of more general disruptions to schooling that arise from the ADHD assessment and diagnosis ‘journey’.

The clinics recorded a large number of protocol deviations in terms of completion of follow-ups within the study protocol timeframe. Only 37% of QbTests in the intervention arm were completed within 2–4 weeks of medication initiation and only 30% of two follow-up contacts in the control arm were completed within the 12-week study timeline. Interviews with the clinicians reflect that the appointment schedules for both arms were challenging for the clinics. The first follow-up was difficult for clinics to adhere to because appointment schedules are often filled at least 4 weeks in advance, leaving very little flexibility for clinicians to schedule the study appointments. Clinicians reported holding some appointments outside their usual clinic times to deal with this problem, but this would not be feasible in a larger trial. One option would be to design a more flexible trial protocol that can be adapted to fit with each service’s appointments system.

Interestingly, findings from the pro forma indicated that early use of the QbTest on medication may be most beneficial, with clinicians making more treatment changes (changing dose or type of ADHD medication) in the experimental arm at follow-up 1 compared to the control group, but little difference at follow-up 2. This may be because the QbTest promoted earlier treatment optimisation, which was also evidenced in the improved SNAP-IV scores and more clinicians reporting the clinical utility of QbTest at follow-up 1 than 2. We are cautious not to over-interpret findings from this small sample; however, this warrants further investigation and suggests that a more naturalistic protocol allowing clinicians to use QbTest according to their clinical judgment (but with a minimum of one pre and post medication test) may be more suitable.

### QbTest and clinic management

Interview data indicated that the objectivity of the QbTest was appreciated by clinicians and parents alike in comparison to informant measures that are traditionally used to monitor medication. Clinicians also commented that it is not feasible to use QbTest in medication management for all cases due to the additional clinic time and resources required, when balanced against the additional information that Qb gives; for more simple cases, it is sufficient to monitor medication using established clinical methods. However, there is perceived value in using QbTest for more complex patients, which includes co-morbidities, and any examples where there is contention about treatment approaches, for which case Qb is particularly helpful. The interview data also indicated that clinicians find QbTest helpful in communication with families, including reaching a shared decision about any particular course of action. These findings relate to normalisation process theory in two ways. Firstly, that normal working practices do not fit with the use of QbTest in every case, due to the additional workload it brings and secondly, alongside this, clinicians do not consider it necessary to use QbTest for every case, but prefer to reserve it for complex cases. These findings have implications for the design of a full RCT and any expectation of the implementation of the intervention post-trial.

### Strengths and limitations

This study is strengthened by the involvement of multiple stakeholders, including PPI and clinicians [31]. These stakeholders were instrumental in designing the protocol in the first stage of the study and in evaluating the feasibility and acceptability of the protocol once data collection ended, including via the post-study workshop described above. These insights have led to the following refinements to the protocol to improve its feasibility and acceptability in a definitive RCT. Firstly, difficulties with recruitment led to a sample size smaller than the target of 60. As identified by stakeholders, extending the time period over which a prior QbTest can be considered a baseline for medication is likely to mitigate these difficulties to some extent. Secondly, the pool of eligible participants is relatively small and so it is likely that a larger trial at a greater number of sites would reduce these recruitment difficulties. Thirdly, a more flexible protocol with a larger timescale for follow-up assessments, also identified by our stakeholders, will enhance feasibility of data collection by enabling clinicians to use QbTest in a more flexible manner and by allowing longer for follow-up assessments to be completed. Fourthly, although not the purpose of a feasibility trial, a definitive efficacy trial should record whether the time of day of follow-up QbTest influenced treatment decision-making plans as test interpretation may differ according to whether the effect of stimulant medication has worn off. A final point to consider is that our qualitative interviews were only conducted with parents as children were unwilling to participate or deemed too young by their parents. Although this is common with this young sample [26], we will work with key stakeholders to refine our methods for gathering insight into the experiences of young people.

### Conclusion

The findings of this study suggest that, with some modifications to the follow-up time periods, the method of data collection, and refinement in the methods for health economics data collection, the protocol may be appropriate for a full clinical trial. The stakeholders judged that with further modifications as described, the protocol would be more feasible to implement and would be clinically beneficial. The findings further suggest that the SNAP-IV would be a useful primary outcome for reflecting change in symptoms between trial arms.

## Supplementary Information

**Additional file 1.** CONSORT 2010 checklist of information to include when reporting a pilot or feasibility trial

## Data Availability

The datasets used and/or analysed during the current study are available from the corresponding author on reasonable request.

## Abbreviations

ADHD: Attention deficit hyperactivity disorder
ASD: Autism spectrum disorder
CGI: Clinical global impressions scale
CHU9D: Child health utility 9D
CSRI: Client service receipt inventory
FDA: US food and drug administration
SDQ: Strengths and difficulties questionnaire
SNAP-IV: Swanson, noland and pelham version IV
NHS: National health service
NICE: National institute for health and care excellence
NPT: Normalisation process theory
PPI: Patient and public involvement
QALYs: Quality-adjusted life-years
RCT: Randomised controlled trial
RUSH: Resource use for health
RUSE: Resource use for education
