# Correction to: Optimising medication management in children and young people with ADHD using a computerised test (QbTest): a feasibility randomised controlled trial

**DOI:** 10.1186/s40814-021-00830-2

**Published:** 2021-04-15

**Authors:** Laura Williams, Charlotte L. Hall, Susan Brown, Boliang Guo, Marilyn James, Matilde Franceschini, Julie Clarke, Kim Selby, Hena Vijayan, Neeta Kulkarni, Nikki Brown, Kapil Sayal, Chris Hollis, Madeleine J. Groom

**Affiliations:** 1 School of Medicine, Division of Psychiatry and Applied Psychology, Institute of Mental Health, University of Nottingham Innovation Park, Triumph Road, Nottingham, NG7 2TU UK; 2 Nottingham Clinical Trials Unit, University of Nottingham, Nottingham, UK; 3 Division of Rehabilitation and Ageing, University of Nottingham, Nottingham, UK; 4 United Lincolnshire Hospitals NHS Trust, Grantham and District Hospital, Grantham, Lincolnshire UK; 5 Department of Community Paediatrics, Medway NHS Foundation Trust, Kent, UK; 6 North East London NHS Foundation Trust, Havering CAMHS, Essex, UK; 7 Leicestershire Partnership NHS Trust, Leicester, UK; 8 Centre for ADHD and Neurodevelopmental Disorders (CANDAL), University of Nottingham, Nottingham, UK; 9 NIHR MindTech Medtech Co-operative, Institute of Mental Health, University of Nottingham Innovation Park, Triumph Road, Nottingham, UK; 10 NIHR Nottingham Biomedical Research Centre, Institute of Mental Health, University of Nottingham Innovation Park, Triumph Road, Nottingham, UK

**Correction to: Pilot Feasibility Stud 7, 68 (2021)**

**https://doi.org/10.1186/s40814-021-00788-1**

Following publication of the original article [1], the authors reported an error in the first name of author three, which reads Sue Brown and should be Susan Brown.

The original article has been corrected.
